# Rational Design of Microfluidic Glaucoma Stent

**DOI:** 10.3390/mi13060978

**Published:** 2022-06-20

**Authors:** Thomas Graf, Gitanas Kancerevycius, Linas Jonušauskas, Patric Eberle

**Affiliations:** 1 Institute of Electrical Engineering, Lucerne University of Applied Sciences, CH-6048 Horw, Switzerland; thomas.graf@hslu.ch; 2 Valsigna GmbH, Via Luganetto 4, CH-6962 Lugano-Viganello, Switzerland; gk@valsigna.swiss (G.K.); lj@valsigna.swiss (L.J.)

**Keywords:** microfluidic stent, glaucoma, flow resistance model, bleb drainage, IOP stabilization

## Abstract

Glaucoma is a common, irreparable eye disease associated with high intraocular pressure. One treatment option is implantation of a stent to lower the intraocular pressure. A systematic approach to develop a microchannel stent meshwork that drains aqueous humor from the anterior chamber of the eye into the subconjunctival space is presented. The stent has a large number of outlets within its mesh structure that open into the subconjunctiva. The development approach includes a flow resistance model of the stent. Local adaption of the stent’s tubular dimensions allows for adjustment of the flow resistance. In this way, an evenly distributed outflow into the subconjunctiva is achieved. We anticipate that microblebs will form at the stent outlets. Their size is crucial for drainage and control of intraocular pressure. An analytical model for bleb drainage is developed based on the porous properties of the subconjunctival tissue. Both models—the stent flow resistance model and the bleb drainage model—are verified by numerical simulation. The models and numerical simulation are used to predict intraocular pressure after surgery. They allow for a systematic and personalized design of microchannel stents. Stents designed in this way can stabilize the intraocular pressure between an upper and lower limit.

## 1. Introduction

Glaucoma is the leading cause of irreversible blindness and causes high healthcare costs as well as impaired quality of life and suffering for those affected [1,2,3]. It is estimated to affect more than 60 million people worldwide, with incidence increasing with age, and is most prevalent in Africa and Asia [1,2,3]. Glaucoma belongs to a group of neurodegenerative eye diseases in which the optic nerve and retinal ganglion cells are damaged, caused by elevated intraocular pressure (IOP) [1]. Therapy aims to reduce IOP and may include medication, laser trabeculoplasty or surgery for the treatment of primary open angle glaucoma (POAG), ocular hypertension (OHT) and normal tension glaucoma patients (NTG) [4,5].

Non-invasive treatments with medications and laser trabeculoplasty may not be sufficiently effective or may be associated with side effects in some patients [4]. Patients with IOP that poses a significant risk for progressive glaucoma damage leading to visual impairment, where non-invasive treatments were not successful, are candidates for surgery [5]. In standard trabeculectomy surgery, the outflow resistance of the aqueous humor (AH) is reduced by opening a channel from the anterior chamber through the sclera into the subconjunctival space [6]. The draining AH forms a bleb in the subconjunctival space. From the bleb, AH drains into the subconjunctiva and is absorbed by the highly vascular tissue. However, the outflow facility may decrease postoperatively due to fibrotic reactions and scarring in the subconjunctiva [4,6,7,8]. Alternatively, a glaucoma drainage device (GDD) can be implanted to assist and control the drainage of AH from the anterior chamber through an artificial tube connection [4,9,10,11,12]. Several GDDs have been developed that drain AH into the subconjunctival space (Xen, Allergan Inc., Dublin, Ireland; ExPress, Alcon Inc., Geneva, Switzerland), Schemm’s canal (iStent *inject*, Glaukos Inc., San Clemente, CA, USA; Hydrus, Alcon Inc., Geneva, Switzerland), suprachoroidal space (CyPass, Alcon Inc., Geneva, Switzerland; Solx Gold Shunt, SOLX Inc., Waltham, MA, USA) or outside (Ahmed glaucoma valve plate AGV PF7, New World Medical Inc., Rancho Cucamonga, CA, USA) [4,5]. Solx Gold Shunt is a microfluidic device that distributes AH into the suprachoroidal space through multiple outlets. However, GDD implantation can also lead to fibrotic reactions and scarring postoperatively, resulting in an increase in IOP again after an initial decrease in IOP [4,13]. In subconjunctival drainage, the morphology of the bleb is critical to its drainage capability into the tissue, and the morphology of the bleb may change after surgery resulting in IOP variations, which has been repeatedly studied for both standard trabeculectomy and GDD implants [14,15,16,17].

Stable AH outflow is critical for IOP lowering and stabilization, which can be impaired by scarring and bleb changes. The aim of this study was to investigate and optimize the fluidic properties of a microfluidic stent meshwork that reduces the risk of fibrosis development by its design and that drains into the subconjunctival space with multiple microblebs for IOP stabilization. A fluidic model was developed, including microbleb and tissue behavior enabling step-by-step personalized design of the microfluidic stent.

It has been shown that the appropriate choice of materials as well as the architecture of the GDDs can affect and reduce the postoperative development of fibrosis and scar formation [13,18,19]. A flexible microfluidic stent meshwork implanted in the subconjunctival space with channel sizes of cellular dimensions has demonstrated significantly less fibrosis development than the AGV PF7 in animal models [18]. Although the basic mechanisms for fibrotic response and capsule formation around glaucoma drainage devices are not clearly understood, Amoozgar et al. suggested that two implant-specific factors give rise to tissue reactions: (1) mechanical stress may develop at the interface between the tissue and the implant (e.g., due to a mismatch of mechanical properties) and (2) the implant disrupts the cellular and vascular system of the tissue [18]. It is hypothesized that the incorporation of these two key features into the design of the microfluidic network reduces the development of fibrotic tissue around the implant.

**Figure 1:**
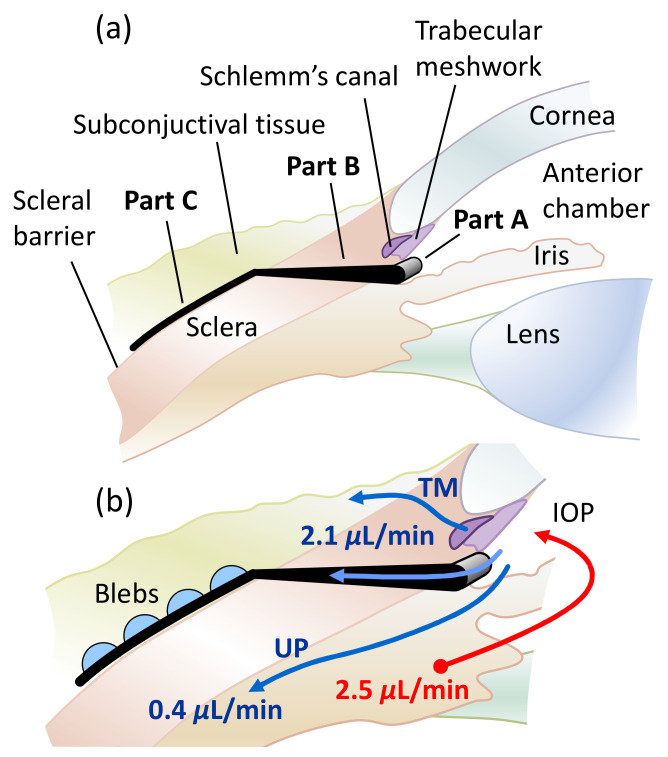
(**a**) Sectional anatomy of the eye with glaucoma stent. Part A is placed in the anterior chamber to collect AH. Part B conducts the AH into the meshwork of part C. Part C drains the fluid into the subconjunctival tissue. (**b**) Drainage pathways and flow balance in the eye after surgery. AH is produced in the ciliary body and drained by the trabecular meshwork (TM) via Schlemm’s Canal, the uveoscleral pathway (UP) and the stent. At the stent outlets, blebs form in the subconjunctiva on the scleral barrier. The indicated flow rates were used in numerical simulation.

**Figure 2:**
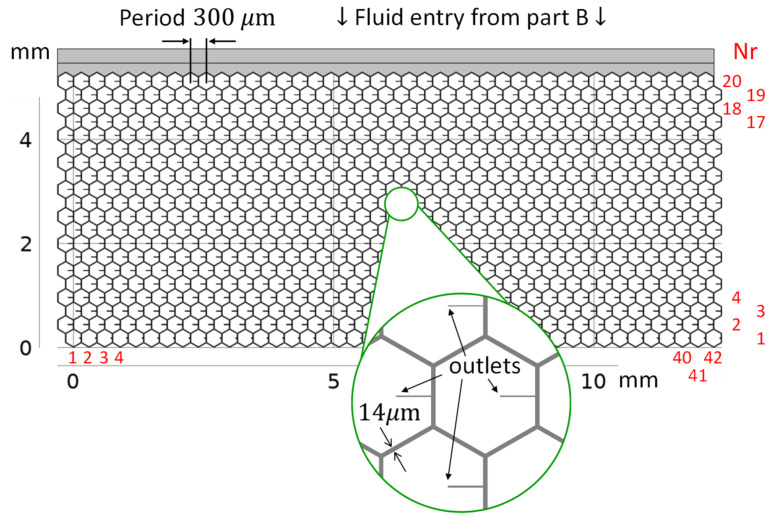
The meshwork consists of honeycomb cells and is 12.6 mm wide and 5.2 mm high. Each hexagonal segment is a microtube (microchannel) with a square internal cross-section 14 μm wide. Each honeycomb cell contains an outlet tube with specific dimensions and a corresponding flow resistance (see enlarged detail). The black numbers are the dimensions; the red numbers denote the columns and rows of the meshwork. The liquid flows evenly from part B above into the meshwork.

Figure 1 schematically shows the placement of the studied microfluidic stent in the eye that drains AH into the subconjunctival space. Part A of the stent collects AH in the anterior chamber of the eye and drains it via part B into part C, a microfluidic mesh. Part C is inserted in the subconjunctiva, on the scleral barrier. The bypass drainage through the stent serves to lower and regulate IOP. It is very important to also include drainage into the subconjunctival tissue and the associated pressure at the stent outlets in the analysis. The outflow of AH into the tissue leads to bleb formation. We argue that microblebs form at each stent outlet, as indicated in Figure 1b. The outflow is uniformly distributed over an area of 0.65 cm^2^ using multiple outlets in the hexagonal meshwork of the current stent design, as shown in Figure 2. The cell-sized dimensions of the microchannels are expected to reduce the risk of fibrosis [18]. IOP can be adjusted by varying the dimensions of the outlet tubes of the stent, while the mesh structure and the dimensions of the mesh channels remain fixed. Large cross-sections are adopted for part A and part B to achieve a near-zero pressure drop until the distribution of AH in Part C. For the meshwork of part C in Figure 2, we used a honeycomb structure with thin connections (channels). The hexagonal geometry provides high in-plane stability and high flexibility for out-of-plane deformations [20]. This preserves the channel dimensions and the uniform outflow of AH into the subconjunctiva, while allowing the mesh to conform to the tissue in the out-of-plane direction at low mechanical stress. In the remainder of the paper, we will discuss only Part C of the stent, which we will refer to simply as the stent or meshwork.

The paper is organized as follows. In Section two, the methods of numerical simulation are briefly explained. Section three describes the models developed to calculate the pressure drop in the stent, including the absorption of the liquid in the subconjunctival tissue. The section concludes with the calculation of IOP after surgery ($IOP_{AS}$) using the current stent design. Section four concludes the present work.

## 2. Numerical Methods

The computational microfluidics of the stent’s tubular mesh (Figure 2) were performed with the software COMSOL Multiphysics 5.6^®^ [21] employing the finite element method (FEM). We used the Laminar Flow interface of the Fluid Flow module and the physical model Creeping Flow. The outflow from the stent openings into the subconjunctival tissue was calculated by means of the Darcy’s Law interface of the Porous Media and Subsurface Flow branch in COMSOL. Finite element meshes were generated automatically using the COMSOL option Physics Controlled Mesh. Typical element size was Finer. COMSOL and the Electrical Circuit interface of the AC/DC module were used to verify the resistor network. We always computed stationary solutions.

## 3. Mathematical Models

An equivalent model with lumped fluidic resistances was developed to calculate the pressure drop across the stent. The resistors were determined from the stent geometry. The model was validated using COMSOL simulations. After that, the role of blebs at the stent outlets was investigated. Drainage of fluid from the bleb into the surrounding subconjunctival tissue was modeled using Darcy’s law for porous media [6]. This was calculated analytically and confirmed by COMSOL simulation. $IOP_{AS}$ is a function of three drainage pathways, including the pressure in the blebs and the pressure drop across the meshwork. The natural outflow via TM, which is typically insufficient in open-angle glaucoma patients, as well as the uveoscleral outflow, were considered in the prediction of the $IOP_{AS}$. They can be treated as parallel flow to the stent outflow [9,22].

### 3.1. Circuit Model of Stent Flow

**Figure 3:**
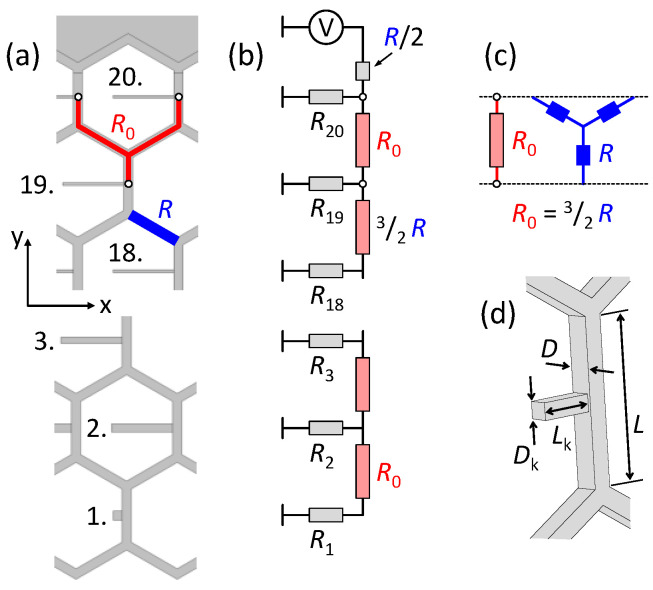
Tubular structure of the stent and fluid resistance model. (**a**) The honeycomb meshwork geometry is the same everywhere; only the outlet tubes have different dimensions. The star-shaped part, colored in red, can be expressed as a fluidic resistance $R_{0}$ between two subsequent outlet tubes. (**b**) Equivalent circuit diagram of a single column of the stent, from the fluid inlet (top) to the individual outlets. The numbered resistors $R_{1}$ to $R_{20}$ correspond to the various outlet tubes along the stent column. Due to the different dimensions, the outlet tubes have distinct flow resistances. The dimensions are chosen so that the same rate of liquid flows out of each tube. The diagram in (**c**) illustrates how the resistance $R_{0}$ can be calculated using the flow resistance R of a straight channel segment (marked in blue). Drawing (**d**) depicts the relevant geometric entities for the calculation of the flow resistance.

Exploiting the symmetries of the hexagonal mesh, the equivalent circuit with lumped-resistances of the flow path along a column (in the negative y-direction) can be derived, see Figure 3. The flow resistance R of a straight hexagon segment can be calculated with the channel dimensions following Hagen–Poiseuille’s law [23]. For a channel with a square cross-section, the equation for the flow resistance is [24]:

(1) $$R≅28.4\cdot \eta \frac{L}{D^{4}}\;\;,$$

where L is the segment length, D is the cross-sectional width of the channel and $\eta ≅7\times 10^{-4\;}\mathrm{Pa}\cdot s$ is the dynamic viscosity of the humorous fluid (≈saline water at 37 °C). The base element of the lumped resistor model, $R_{0}$ connecting two outlet tubes of consecutive rows, can be modeled by a star connection (see Figure 3c) whose contact resistance is $R_{0}=\frac{3}{2}R$ (for details see Appendix A). The contact resistance therefore is

(2) $$R_{0}=42.7\cdot \eta \frac{L}{D^{4}}\;\;.$$

For the squared outlet tube, the flow resistance $R_{k}$ at row k is calculated as in Equation (1):

(3) $$R_{k}≅28.4\cdot \eta \frac{L_{k}}{{\left(D_{k}\right)}^{4}}\;\;,$$

where $L_{k}$ is the tube length and $D_{k}$ is the cross-sectional width of the outlet tube, as shown in Figure 3d. The sequence of connecting resistors $R_{0}$ and outlet resistors $R_{k}$ is the equivalent circuit of the flow path along a column in the mesh (in the negative y-direction) shown in Figure 3b. COMSOL simulations showed that boundary corrections in the first and the last column (out of 42 columns) of the network can be omitted without loss of accuracy. To obtain the same outflow ${\dot{Q}}_{t}$ from each outlet tube, the flow rate to row $\left(k-1\right)\;$ must be $\left(k-1\right){\dot{Q}}_{t}$. This can be ensured if the resistance $R_{k}$ of each outlet tube is $\left(k-1\right)R_{rest\left(k-1\right)}$, where $R_{rest\left(k-1\right)}$ is the flow resistance of the circuit below row k. $R_{k}$ can thus be expressed in terms of $R_{0}$ and $R_{1}$. Examples of $R_{1}$ to $R_{5}$ are given in Table 1. The general equation of the outlet tube resistance in row k is

(4) $$R_{k}=\left(k-1\right)R_{rest\left(k-1\right)}=\frac{1}{2}k\left(k-1\right)R_{0}+R_{1}\;\;.$$

The total resistance of a honeycomb stent column (compare to Figure 3a,b) is thus

(5) Rc=R2+RnrRrestnr−1Rnr+Rrestnr−1=R03+RnrRnrnr−1Rnr+Rnrnr−1=nr2−16R0+R1nr  ,

where $n_{r}$ is the number of honeycomb rows. The term R/2 on the left of Equation (5) stems from the short, straight channel segment between stent part B and the first outlet (see Figure 3). The correctness of Equation (5) has been verified with a simulation using the Circuit Interface of the AC/DC module of COMSOL Multiphysics 5.6^®^ [21]. The lowest resistor $R_{1}$ contributes little to the total resistance and is a free design parameter. It shall be noted that the channels below the lowest outlet tube in row 1 (below resistance $R_{1}$) are preferably closed to avoid stagnant liquid.

AH production rate varies among individuals and was found to slightly decrease with age and to be relatively independent of IOP (see Appendix B for details, also regarding POAG/OHT patients). In the 20- to 83-year age group, it was reported as 2.4±0.6 μL/min (mean ± SD) during daytime, with diurnal variations: morning ≈3.0 μL/min, afternoon ≈2.4 μL/min and night ≈1.5 μL/min [25]. We set the production rate to $\dot{Q}=2.5\;\mu L/\min$ and then accounted for the diurnal variation of the rate in the analysis of $IOP_{AS}$. The presented model for designing stents can be easily personalized to other production rates by setting $\dot{Q}$ in the step-by-step procedure presented in Appendix C. Part of the AH is drained via TM and the other part via UP [6,26]. Reported fractions of uveoscleral outflow vary between 4 and 54% and depend not only on biological factors but also on the measurement method (see Appendix B for details) [25,27]. We set uveoscleral outflow ${\dot{Q}}_{up}$ at 15% of total outflow (${\dot{Q}}_{up}=0.15\cdot \dot{Q}=0.4\frac{\mu L}{\min}$). The flow rate through the stent ${\dot{Q}}_{s}$ ranges from 1.5 to 1.8μL/min. It depends on the fluid resistance of TM and on IOP, as explained in Section 3.4 and Appendix C. In the following computations we used a typical ${\dot{Q}}_{s}=1.7\;\mu L/\min$. For the stent shown in Figure 2, the values in Table 2 were obtained. The dimensions of the stent meshwork and of the lowest outlet tube (resistance $R_{1}$) were chosen to reach $IOP_{AS}=14\;\mathrm{mmHg}$ (see Section 3.4). The latter is a free design parameter of the stent and is selected within a healthy range. It is important to understand that the dimensions of the channel cross-sections must be maintained very precisely in order to achieve the targeted resistance value of the stent (or the targeted $IOP_{AS}$). Equations (1) and (2) show that the cross-sectional width of the tube enters with the fourth power. Small dimensional variations have a large effect. Consequently, the manufacturing process of the stent must be of high precision and reproducibility.

### 3.2. Numerical Model of Stent Flow

The lumped resistance model was validated by computational fluid dynamics with the software COMSOL Multiphysics^®^ [21]. Since the flow in the stent meshwork is in the low Reynolds regime Re≪1, it is a so-called Stokes flow for which the physical model Creeping Flow from COMSOL can be used. This physics omits the inertial term in the Navier Stokes equation: $\varrho \left(u\cdot \nabla \right)u=0$, where ϱ is the fluid density, u the fluid velocity and ∇ the Nabla operator. Tests with the fully laminar formulation did not show any differences. Computation time in this case was, however, 30% longer.

**Figure 4:**
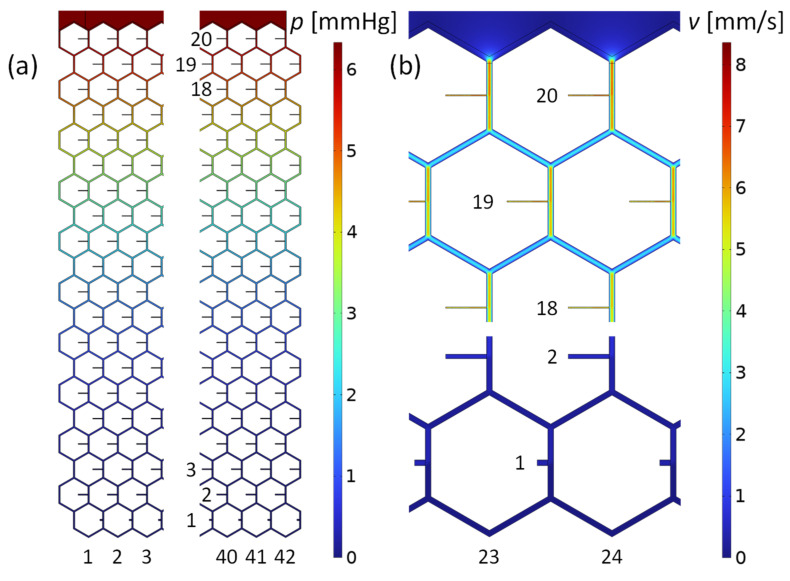
Pressure and flow velocity field in the microchannel mesh computed with the program COMSOL. The boundary conditions were the inlet flow rate of $1.7\frac{\mu L}{\min}$ and the outlet pressure of 0 mmHg at each orifice. (**a**) Pressure distribution in the stent of the leftmost and rightmost part of the mesh. The maximum value is 6.3 mmHg at the inlet, above row 20. (**b**) Flow velocities in the midplane of the meshwork. Shown are flow details at top and bottom of the stent, along the centerline of the honeycomb structure.

The last two rows of Table 2 show that the circuit calculation of the pressure across the stent using the lumped resistor model agrees to within 5% with the COMSOL simulation. Furthermore, numerical simulation showed that the outflow at each stent opening is ${\dot{Q}}_{t}=2.0\pm 0.4\frac{\mathrm{nL}}{\min}$ consistent with ${\dot{Q}}_{s}$ divided by the number of stent outlets. The lowest outlet row, however, gives a slightly higher outflow, independent of the mesh refinement. This is a consequence of the unfavorable length-to-diameter ratio of the lowest outlet tubes where Hagen–Poiseuille’s law for long narrow tubes no longer applies. Furthermore, the remaining flow rate in the lowest tubes is very small and numerical uncertainty increases. Other, small variations in outflow are random and due to numerical inaccuracies. Smaller FEM mesh sizes result in similar flow variations at the individual openings and can be considered “numerical noise”. The integral of the velocity field across all orifices yields a total outflow of $1.7\frac{\mu L}{\min}$. This is equal to the total inflow, which is a boundary condition and demonstrates consistency. Figure 4 shows the pressure drop across the stent mesh and the velocity field in the midplane of the stent channels. The highest velocity of $7\frac{\mathrm{mm}}{s}$ occurs in the center of the channels at the inlet to the meshwork. The velocity in the center of the lowest channel is 0.3 mm/s. In summary, the numerical flow simulation confirmed our lumped resistance model with high accuracy.

### 3.3. Model of Drainage to Subconjunctival Tissue

The AH drains from the stent into the surrounding, highly vascular subconjunctival tissue. Blebs form in the subconjunctival space at the outlets of the stent [4]. The pressure at the outlets is also the pressure in the bleb and is determined by the absorption properties of the porous subconjunctival tissue around the bleb, and the size and shape of the bleb. The microscopic capillaries in the subconjunctiva with their complex ramification and varying sizes can be transferred into a homogeneous model using Darcy’s law that emulates the complex microvascular structure [6]. We used COMSOL Multiphysics^®^ to compute Darcy’s law inside the tissue. The FEM program solves the following equation for the pressure p:

(6) $$\varrho \frac{\kappa }{\eta }\Delta p-S_{p}p=0\;\;,$$

where Δ is the Laplace operator, ϱ is the density of the fluid, κ is the fluid permeability in the subconjunctival tissue and η is the dynamic viscosity of the fluid (saline water). The ratio $K=\frac{\kappa }{\eta }$ is referred to as hydraulic conductivity [6]. $S_{p}p$ corresponds to the source term in Darcy’s law for fluid removal through or fluid entry into the blood capillaries in the tissue. The constant $S_{p}$ can be expressed as $S_{p}=\varrho \cdot L_{p}\frac{S_{A}}{V}$, where $L_{p}$ and $\frac{S_{A}}{V}$ are the hydraulic permeability of the blood vessel walls and the surface area of the vessel walls per tissue volume, respectively [6].

The pressure p of the source term in Equation (6) is composed of the hydrostatic and oncotic pressure differences between capillaries and interstitium. It can be derived from Starling’s law as $p=\left(p_{c}-p_{i}\right)\;-\sigma \left[\pi_{c}-\;\pi_{i}\right]$, where $p_{c,i}$ are the hydrostatic and $\pi_{c,i}$ the oncotic capillary and interstitium pressures and σ is the reflection coefficient [6,28]. For our numerical simulations and analytical models, we used the generally accepted subconjunctival properties given in Appendix D. For normal tissue, $p_{c}-\sigma \left[\pi_{c}-\;\pi_{i}\right]\approx 0$, yielding $p=-p_{i}$, which is the dependent variable solved for [6,29].

The meshwork is implanted between the subconjunctiva and the sclera during the operation. The subconjunctival tissue closes onto the surface of the meshwork during healing. An initial bleb with the extension of the meshwork or larger is likely to form, with low drainage resistance and low bleb pressure during the early postoperative phase [4]. As healing continues, the subconjunctiva closes onto the individual outlets of the stent. Microblebs form. This presumably completes the healing process. An analytical model of drainage of a single microbleb within absorbent tissue is developed and numerically validated in Section 3.3.1. A simulation of a microbleb array in the subconjunctiva is presented in Section 3.3.2.

#### 3.3.1. Drainage from Hemispherical Microbleb

Microblebs presumably form around each outlet orifice of the stent in the course of healing. We assume hemispherical microblebs, although morphological observations of macroblebs have revealed different, non-spherical shapes [15,16,17]. We justify our assumption by the following facts: (i) The stent is resting between the subconjunctival tissue and the scleral barrier, which is assumed impermeable. Hydraulic conductivity of the sclera is significantly smaller than that of the subconjunctival tissue (≈ by a factor 50), thus the sclera is modeled as impermeable. (ii) The stent has multiple outlets with sufficient separation between them and the flow rate per outlet is very small, ${\dot{Q}}_{t}=2.0\frac{\mathrm{nL}}{\min}$ (see Table 2). (iii) The fine stent structure facilitates tissue overgrowth and causes only little disruptions in the tissue structure, which has been shown to reduce the risk of fibrosis and scar development [18]. Low fibrosis and little scarring has the advantage that the fluid permeability of the tissue stays high and does not change over time. Drainage remains predictable and more stable. A healthy subconjunctival tissue ensures long-term stability of IOP in the operated eye. For the reasons (i) to (iii), hemispherical microblebs are likely to form around each outlet orifice.

In the following, we calculate the pressure in a hemispherical bleb due to the drainage resistance in the subconjunctival tissue. In spherical coordinates and exploiting spherical symmetry, the governing Equation (6) becomes

(7) $$\frac{1}{r^{2}}\frac{d}{dr}\left(r^{2}\frac{d}{dr}p\right)-\frac{S_{p}}{\varrho }\frac{\eta }{\kappa }p=0\;\;,$$

whereby the angle dependence disappears: $\frac{d}{d\phi }=\frac{d}{d\theta }=0$. The r is the distance from the coordinate origin. Equation (7) can be rewritten as $r\frac{d^{2}}{dr^{2}}p+2\frac{d}{dr}p-\frac{S_{p}}{\varrho }\frac{\eta }{\kappa }rp=0$. With the substitution q=r·p this equation becomes $\frac{d^{2}}{dr^{2}}q-\frac{S_{p}}{\varrho }\frac{\eta }{\kappa }q=0$ from which follows the solution of Equation (7) as

(8) $$p\left(r\right)=\frac{B}{r}e^{-\sqrt{C}\cdot r}\;,\;$$

where $C=\frac{S_{p}}{\varrho }\frac{\eta }{\kappa }=2.0\times 10^{6}\frac{1}{m^{2}}$ (see above) and B is a constant to be determined from the flow rate ${\dot{Q}}_{t}$ of the outlet tube into the bleb. The quantity $1/\sqrt{C}=0.7\;\mathrm{mm}$ can be considered as the characteristic drainage length. Equation (8) satisfies the boundary condition that the fluid pressure is zero far from the bleb: $p\left(\infty \right)=0$. Darcy’s law and spherical symmetry provide the equation for the radial flow velocity $u_{r}$ inside the vascular tissue:

(9) $$u_{r}=-\frac{\kappa }{\eta }\nabla_{r}p\to u_{r}=\frac{\kappa }{\eta }B\left(\frac{\sqrt{C}}{r}+\frac{1}{r^{2}}\right)e^{-\sqrt{C}\cdot r}\;\;,$$

where $\nabla_{r}=\frac{d}{dr}$ is the gradient operator in radial direction. From the bleb, the flow ${\dot{Q}}_{b}$ drains into the surrounding tissue and is absorbed there. ${\dot{Q}}_{b}$ is equal to ${\dot{Q}}_{t}$ in the outlet tube of the stent. ${\dot{Q}}_{b}$ relates to the velocity $u_{b}$ at the hemispherical surface of the bleb of the radius $r_{b}$ in the following way:

(10) $$u_{b}=\frac{{\dot{Q}}_{b}}{2\pi \;r_{b}^{2}}\;\;.$$

Note the factor of 2 instead of 4 in Equation (10), which accounts for drainage through half of a spherical shell. The hemispherical bleb is assumed to rest on the impermeable scleral barrier and there is no drainage in that direction. Combining Equations (9) and (10) in $u_{b}=u_{r}\left(r_{b}\right)$ yields the constant B:

(11) $$B=u_{b}\frac{\eta }{\kappa }\frac{1}{\left(\frac{\sqrt{C}}{r_{b}}+\frac{1}{r_{b}^{2}}\right)e^{-\sqrt{C}\cdot r_{b}}}=\frac{{\dot{Q}}_{b}}{2\pi }\frac{\eta }{\kappa }\frac{1}{\left(r_{b}\sqrt{C}+1\right)e^{-\sqrt{C}\cdot r_{b}}}\;\;.$$

Equation (11) inserted into Equation (8) yields the pressure at the bleb surface, that is, where the fluid enters the vascular subconjunctival tissue:

(12) $$p\left(r_{b}\right)=p_{b}=\frac{{\dot{Q}}_{b}}{2\pi }\frac{\eta }{\kappa }\frac{1}{\left(r_{b}^{2}\sqrt{C}+r_{b}\right)}\;\;.$$

**Figure 5:**
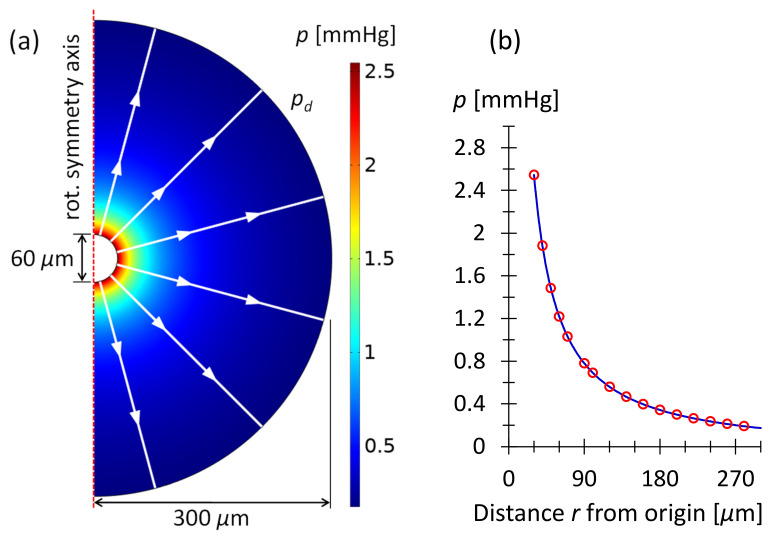
(**a**) COMSOL simulation of the outflow from a bleb with radius $r_{b}=30\;\mu m$. The inlet boundary condition is the mass flow rate $\dot{M}={\dot{Q}}_{b}\cdot \varrho =3.3\times 10^{-11}\frac{\mathrm{kg}}{s}$ at the hemispherical bleb surface. This corresponds to ${\dot{Q}}_{b}={\dot{Q}}_{t}=2.0\frac{\mathrm{nL}}{\min}$, given in Table 2. The outer boundary condition is the pressure $p_{d}=0.17\;\mathrm{mm}\;\mathrm{Hg}$ at the domain boundary at $r_{d}=300\;\mu m$. $p_{d}$ is calculated by means of Equations (8) and (11) and used to mimic an infinite domain. The white lines and arrows indicate the flow direction given by the simulation. The axis of rotational symmetry is shown as a vertical, dashed red line. (**b**) Pressure as a function of distance from the origin. The blue line is the COMSOL result; the red circles are obtained using Equations (8) and (11).

Equations (8)–(12) were verified by means of COMSOL computations. Figure 5 shows the pressure obtained by numerical simulation in the subconjunctival tissue outside a bleb with radius $r_{b}=30\;\mu m$. In Figure 5b, the numerical result is compared to the analytical solution of Equation (8). The figure shows that both methods of calculating pressure as a function of distance from the origin are in exact agreement.

If all stent outlet tubes empty into a hemispherical bleb the stent pressure increases by the pressure of Equation (12). In other words, the flow resistance $R_{b}$ of the bleb drainage must be added to each outflow resistance of the stent. The drainage resistance from the bleb to the tissue is calculated as follows:

(13) $$R_{b}=\frac{p_{b}}{{\dot{Q}}_{b}}=\frac{\eta }{2\pi \;\kappa }\cdot \frac{1}{r_{b}\left(r_{b}\sqrt{C}+1\right)}≅\frac{\eta }{2\pi \;\kappa \;r_{b}}\;\;.$$

The last term of Equation (13) is an approximation that applies with an accuracy of 10% for bleb radii $r_{b}<70\;\mu m$, i.e., $r_{b}\sqrt{C}<0.1$. The drainage resistance is inversely proportional to the radius of the bleb. If the bleb shrinks and its radius reduces to the radius of the outlet tube (≈10 μm), the bleb drainage resistance becomes $R_{b}≅4\;\mathrm{mmHg}\cdot \min/\mathrm{nL}$.

A hemispherical perforated shell can be produced at each outlet as part of the stent. This would define the minimum size of the bleb and limit the maximum drainage resistance. Thus, the upper IOP limit can be set by the stent design.

#### 3.3.2. Drainage from Bleb Array

**Figure 6:**
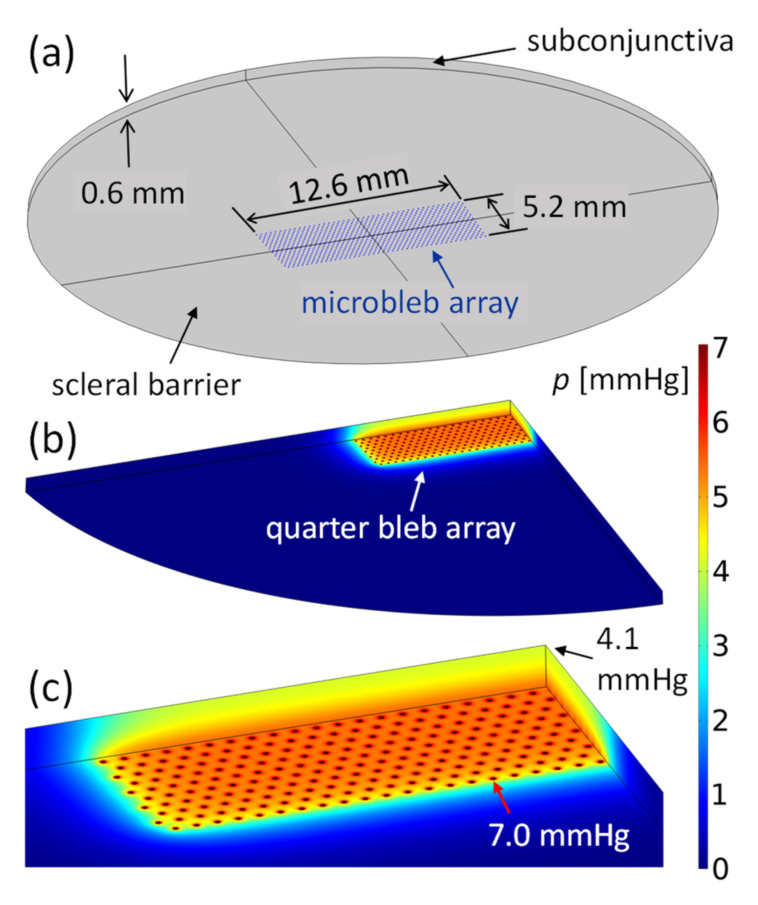
(**a**) Microbleb array of $12.6\times 5.2{\;\mathrm{mm}}^{2}$ of the current stent meshwork. The microblebs lie on the scleral barrier and are spaced 300 µm apart. All blebs have a radius of $r_{b}=30\;\mu m$. AH flows from the bleb surfaces into the 0.6 mm thick subconjunctiva. (**b**,**c**) Drainage pressure field in the subconjunctival tissue determined by COMSOL simulation. The total flow rate of the bleb array is $1.7\frac{\mu L}{\min}$. Figure 6b shows a quarter of the simulation domain. Figure 6c is an enlarged view of 6b. The pressure inside the blebs is 7 mmHg and reaches 4.1 mmHg at the surface of the subconjunctiva. The pressure decreases rapidly in the plane of the array within the characteristic length $1/\sqrt{C}=0.7\;\mathrm{mm}$.

The hexagonal structure of the present stent meshwork results in the hexagonal array of 42×20 blebs depicted in Figure 6a. The bleb array is situated on the scleral barrier, below the 0.6 mm thick subconjunctiva. The hemispherical bleb surfaces empty AH into the adjacent subconjunctival tissue. Equation (13) of the bleb drainage resistance applies to a single bleb embedded in extended absorbent tissue. The proximity of other blebs in the array of Figure 6 and the limited thickness of the subconjunctiva increase the drainage resistance of the blebs. Equation (13) must be considered as the lowest possible bleb drainage resistance. We used Darcy’s law (Equation (6)) and the characteristic tissue values given in Appendix D to calculate the drainage resistance of a bleb array. Figure 6b,c shows the pressure in the subconjunctival tissue obtained by COMSOL simulation. All hemispherical blebs in the hexagonal arrangement of Figure 6 have a radius $r_{b}=30\;\mu m$ and are separated from each other by $d_{a}=300\;\mu m$. The constant pressure at the bleb surfaces divided by the flow rate of AH through the stent and the bleb surfaces yields the array drainage resistance. Drainage resistance is independent of the flow rate (Equation (6) is a linear partial differential equation). The array drainage resistance $R_{a}$ is used to calculate $IOP_{AS}$ in Section 3.4.

**Figure 7:**
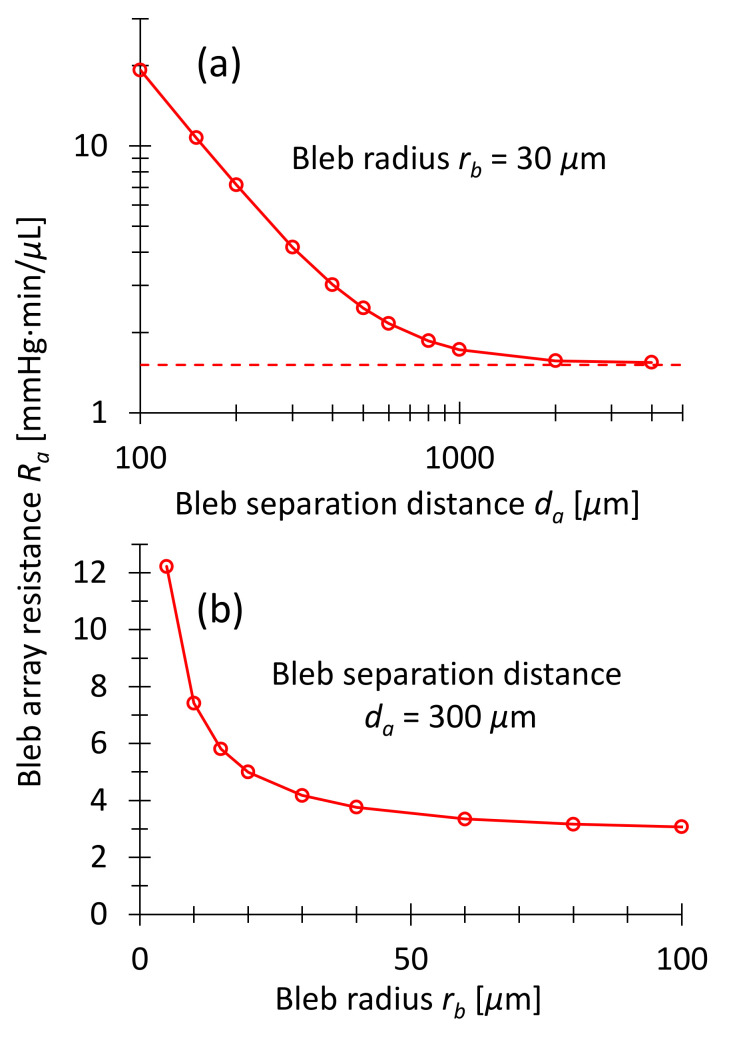
Drainage resistance $R_{a}$ obtained by COMSOL simulations of hexagonal arrays as in Figure 6. (**a**) $R_{a}$ as a function of bleb spacing $d_{a}$. The bleb radius is constant $r_{b}=30\;\mu m$. The curve reaches the value of $1.5\;\mathrm{mmHg}\frac{\min}{\mu L}$ for large separations, which is consistent with Equation (13) for free blebs. (**b**) $R_{a}\;$ as a function of bleb radius $r_{b}$. The bleb spacing is constant at $d_{a}=300\;\mu m$. For bleb radii $r_{b}>30\;\mu m$, the curve terminates in a constant value corresponding to the drainage resistance of a shallow macrobleb of $12.6\times 5.2{\;\mathrm{mm}}^{2}$ surface area.

Figure 7a shows the array resistance $R_{a}$ as a function of bleb separation distance $d_{a}$. All bleb radii are equal to $r_{b}=30\;\mu m$ in Figure 7a. At large separation drainage resistance of the array approaches the resistance of an isolated bleb according to Equation (13), divided by the number of blebs in the array: $R_{a}\left(d_{a}\to \infty \right)=\frac{R_{b}\left(r_{b}=30\mu m\right)}{42\cdot 20}=1.5\;\mathrm{mmHg}\frac{\min}{\mu L}$. The horizontal line in Figure 7a indicates this lower limit.

Figure 7b shows $R_{a}$ as a function of bleb radius $r_{b}$ at constant bleb spacing $d_{a}=300\;\mu m$. The array drainage resistance approximates the value of a single, shallow macrobleb covering the entire stent outlet area of $12.6\times 5.2\;{\mathrm{mm}}^{2}$ when the blebs overlap completely. Figure 7b demonstrates that for bleb radii larger than 30 μm the array drainage resistance is almost constant between 4 and 3 mmHg·min/μL. This allows the IOP to be set relatively precisely, regardless of the bleb diameter, as long as it does not fall below a minimum size. The minimum diameter can be integrated in the stent design, as explained at the end of Section 3.3.1.

### 3.4. IOP after Surgery

**Figure 8:**
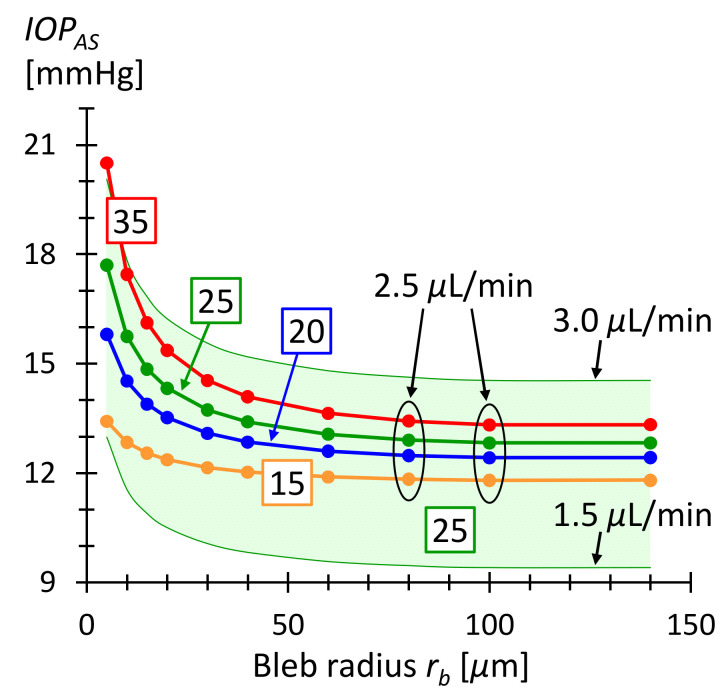
$IOP_{AS}$ as a function of microbleb radius $r_{b}$. The bleb separation is $d_{a}=300\;\mu m$ corresponding to the geometric period in the current honeycomb design. The numbers in the boxes are the IOP values before surgery in units of mmHg. The curves were calculated using Equation (16) and $\dot{Q}.$ $IOP_{AS}$ ranges from 12 and 15 mmHg for bleb radii greater than 30 μm. The green shaded area corresponds to the $IOP_{AS}$ for AH production rate varying between 1.5 and 3.0 μL/min and an IOP before surgery of 25 mmHg. The green shaded area demonstrates that the IOP is within a healthy range even with daily fluctuating production rates [4].

By combining the equations and findings above, the effectiveness of the stent in lowering $IOP_{AS}$ can be analyzed. In a first step, the flow resistance of TM $R_{TM}$ of the diseased eye is determined using the Goldmann equation:

(14) $$\dot{Q}=\frac{IOP_{BS}-p_{EV}}{R_{TM}}+{\dot{Q}}_{UP}\;\;,$$

where$\;IOP_{BS}$ is the IOP before surgery and $p_{EV}=11\;\mathrm{mmHg}$ is the episcleral vein pressure. The resistance $R_{TM}$ can be determined by solving Equation (14) using the known $IOP_{BS}$. In the second step, $IOP_{AS}$ is calculated by extending the Goldmann Equation (14) with the additional term ${\dot{Q}}_{S}=\frac{IOP_{AS}}{R_{sa}}\cdot$ for the stent flow rate:

(15) $$\dot{Q}=\frac{IOP_{AS}-p_{EV}}{R_{TM}}+\frac{IOP_{AS}}{R_{sa}}+{\dot{Q}}_{UP}$$

(16) $$IOP_{AS}=\frac{\dot{Q}-{\dot{Q}}_{UP}+\frac{p_{EV}}{R_{TM}}}{\frac{1}{R_{TM}}+\frac{1}{R_{sa}}}=\frac{\left(\dot{Q}-{\dot{Q}}_{UP}\right)\cdot R_{sa}\cdot IOP_{BS}}{\left(\dot{Q}-{\dot{Q}}_{UP}\right)\cdot R_{sa}+IOP_{BS}-p_{EV}}\;\;,$$

where $R_{sa}$ is the total flow resistance from stent entry to drainage in the tissue. $R_{sa}=R_{s}+R_{a}$ is the series resistance of the stent resistance $R_{s}=R_{c}/42$ (see Table 2) and the bleb array resistance $R_{a}$ (see Figure 7). The current stent consists of 42 columns and 20 rows. Equation (16) assumes that $p_{EV}$ is the same in the diseased and healthy eye. Figure 8 shows $IOP_{AS}$ as a function of bleb radius, calculated with Equation (16) for various $IOP_{BS}$. For bleb radii larger than 30 μm the postoperative $IOP_{AS}$ is between 12 and 15 mmHg for preoperative $IOP_{BS}$ ranging from 20 to 35 mmHg. A bleb size of r=30 μm presumably disrupts the surrounding tissue little [18]. The microbleb size is then of the order of a few cell diameters. This implies that fibrosis is unlikely and scars are also unlikely to form. This in turn means that the subconjunctival absorption properties remain unchanged and drainage into the tissue does not alter. Consequently, $IOP_{AS}$ should remain constant over time. The higher limit of bleb size is given by the distance between them and possible overlaps, but also by the available space in the subconjunctiva. In case the microblebs overlap and form a larger macrobleb, the pressure in the bleb could become nearly zero. The lower IOP limit can be adjusted in this case by the pressure drop across the stent to prevent hypotony.

### 3.5. Study Limitations

The model presented in this study has the following limitations. Published and accepted values were used for the properties of the subconjunctival tissue. They are associated with uncertainties or may change postoperatively due to wound healing and/or medication [6,14]. The trabecular flow resistance $R_{TM}$ was treated as constant in the model, i.e., as a linear flow-pressure relation through TM. $R_{TM}$ may increase at higher IOP, partly due to the collapse of Schlemm’s canal [9]. However, studies have shown that clinical observations can be reproduced with the aforementioned limitations of the model [6,22]. Finally, the meshwork-based stent under investigation, including the presumed formation of microblebs, has not been tested in vitro or in vivo.

## 4. Conclusions

The proposed stent can effectively lower elevated IOP to a level in the target range between 10 and 15 mmHg [4]. Outflowing AH is uniformly distributed over an area of $0.65\;{\mathrm{cm}}^{2}$ into the subconjunctival tissue via multiple microblebs. We find that IOP is almost insensitive to microbleb size variations ($\Delta IOP_{AS}\;≲\;1\;\mathrm{mmHg}$ for $30\;\mu m\leq r_{b}\leq 150\;\mu m$). Lower and upper IOP can be limited with the stent design. The lower limit is determined by the stent resistance if the bleb is large. The upper limit can be set with a perforated shell at each stent outlet, defining the minimum bleb size. The computational models presented can predict IOP after stent implantation. The models enable a step-by-step procedure for a personalized stent production (see Appendix C).

A prerequisite for personalized production is a reproducible and high-precision manufacturing process. The femtosecond laser-induced 2-photon photopolymerization (2PP) technology meets these conditions and guarantees the necessary precision. It combines printing resolution down to hundreds of nm with throughput rivaling micro stereolithography (µSLE) at sub-mm and mm scales [30]. It allows using various biocompatible polymers, assuring superb biocompatibility and adequate mechanical properties needed for medical applications [31,32].

It should be mentioned that the outlet resistances from the hexagonal stent meshwork can also be realized in ways other than straight, narrow and perpendicular outlet tubes. For example, the tubes could be adhered to the main channels. Or a thicker outlet tube with internal resistance could be fabricated. Or a meandering design could be chosen.

In vitro or in vivo study of the presented microfluidic meshwork stent would be the next step from a technological and medical point of view. Of particular interest is the careful study of microbleb formation and its effect on IOP.

Finally, the introduced approach could also be transferred to other stent designs and applications.

## Figures and Tables

**Table 1:** Equations to calculate the outlet tube resistances as a function of the connecting resistance $R_{0}$ and the bottom outlet resistance $R_{1}$. Only the equations for the lowest outlet tubes are shown in the table. The general equation for the outlet resistance of row k is presented in the text (Equation (4)). $R_{rest\left(k-1\right)}$ is the flow resistance of the circuit below row k.

Row k	Resistance of Outlet Tube in Row k	Resistance of Circuit Below Row k
1	$R_{1}$	
2	$R_{2}=R_{rest1}=R_{0}+R_{1}$	$R_{rest1}=R_{0}+R_{1}$
3	$R_{3}=2R_{rest2}=2\left(R_{0}+\frac{R_{2}R_{rest1}}{R_{2}+R_{rest1}}\right)=2\left(R_{0}+\frac{1}{2}R_{rest1}\right)=3R_{0}+R_{1}$	$R_{rest2}=\frac{3}{2}R_{0}+\frac{1}{2}R_{1}$
4	$R_{4}=3R_{rest3}=3\left(R_{0}+\frac{R_{3}R_{rest2}}{R_{3}+R_{rest2}}\right)=3\left(R_{0}+\frac{2}{3}R_{rest2}\right)=6R_{0}+R_{1}$	$R_{rest3}=\frac{6}{3}R_{0}+\frac{1}{3}R_{1}$
5	$R_{5}=4R_{rest4}=4\left(R_{0}+\frac{R_{4}R_{rest3}}{R_{4}+R_{rest3}}\right)=4\left(R_{0}+\frac{3}{4}R_{rest3}\right)=10R_{0}+R_{1}$	$R_{rest4}=\frac{10}{4}R_{0}+\frac{1}{4}R_{1}$

**Table 2:** Resistances, flow rates and pressures of the meshwork of Figure 1 and Figure 2. The resistances R and $R_{1}$ are obtained from the channel geometry. The stent flow rate ${\dot{Q}}_{s}$ is an example value after surgery. The pressure difference $p_{s}$ across the stent is then calculated with the stent fluid model using the mentioned quantities or with COMSOL simulations using the specified geometry.

R	$9.0\times 10^{13}$ 11.2	$\mathrm{Pa}\cdot s/m^{3}$ mmHg·min/μL	Equation (1)	D×D×L=14 μm×14 μm×173 μm
$R_{0}$	$1.35\times 10^{14}$ 16.8	$\mathrm{Pa}\cdot s/m^{3}$ mmHg·min/μL	Equation (2)	
$R_{1}$	$1.35\times 10^{13}$ 1.69	$\mathrm{Pa}\cdot s/m^{3}$ mmHg·min/μL	Equation (3)	$D_{1}\times D_{1}\times L_{1}=14\;\mu m\times 14\;\mu m\times 26\;\mu m$
$\;R_{c}$	$1.33\times 10^{15}$ 166	$\mathrm{Pa}\cdot s/m^{3}$ mmHg·min/μL	Equation (5)	Resistance of a whole column composed of 20 rows
$R_{s}$	$1.16\times 10^{13}$ 3.95	$\mathrm{Pa}\cdot s/m^{3}$ mmHg·min/μL	$\frac{R_{c}}{42}$	Resistance of a whole stent meshwork composed of 20 rows and 42 columns
${\dot{Q}}_{s}$	1.7	$\frac{\mu L}{\min}$	specified	typical stent flow rate
${\dot{Q}}_{c}$	40	$\frac{\mathrm{nL}}{\min}$	$={\dot{Q}}_{s}/42$	Flow rate of a whole column of 20 outlet tubes
${\dot{Q}}_{t}$	2.0	$\frac{\mathrm{nL}}{\min}$	$={\dot{Q}}_{s}/\left(42\times 20\right)$	Flow rate of a single outlet tube
$p_{s}\left\{\mathrm{model}\right\}$	6.7	mmHg	$=R_{c}\cdot {\dot{Q}}_{c}=R_{s}\cdot {\dot{Q}}_{s}$	
$p_{s}\left\{\mathrm{COMSOL}\right\}$	6.3	mmHg	COMSOL simulations	Section 3.2. and Figure 4

**Figure A1:**
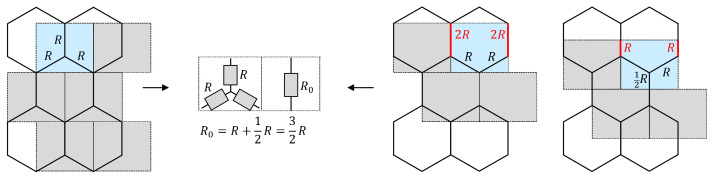
Various possible unit cells of the tubular hexagonal mesh are shown as shaded rectangles. The total resistance *R*_0_ in each unit cell is the same.

## Appendix A. Calculation of Resistance $R_{0}$

In the drawing below, the shaded rectangles are the unit cells (repeating blocks) of the stent. The resistance within a rectangle is also the resistance $R_{0}$ between two successive outlet tubes along a stent column. The resistance of a rectangle does not change when it is moved, considering that the resistance of a mesh tube doubles (in red) when it is shared among two cells. The stent resistance may not depend on the choice of the unit cell. The resistance of each of the three highlighted rectangles is $R_{0}=\frac{3}{2}R$.

## Appendix B. Aqueous Humor Production Rate and Uveoscleral Outflow

AH production rate is individual for each person and depends on several factors. It was found to be 2.4±0.6 μL/min (mean ± SD) over the daytime in the age group 20–83 years, although 2.63±0.63 μL/min was also reported [25,33]. The rate varies diurnally and is usually about 3.0 μL/min in the morning, 2.4 μL/min in the afternoon, and drops to 1.5 μL/min at night [25]. With increasing age, the rate decreases slightly, falling at approximately 3.2% per decade from the age of 10, with no gender differences observed [33]. However, IOP does not usually decrease with age-related decline in humor production because of the counteracting increase in outflow resistance of TM with increasing age [34]. AH production rate was reported to be insensitive to moderate IOP changes [25,33,35]. White individuals affected by POAG or OHT were found to have similar production rates (2.36±0.63 μL/min) compared to the healthy control group (2.19±0.47 μL/min), while in black subjects, the healthy control group had a lower rate (1.81±0.53 μL/min) than POAG/OHT affected patients (2.35±0.53 μL/min) [35]. POAG patients are the potential group for glaucoma surgery but the humor rate data from the aforementioned study was obtained from a rather limited number of participants (POAG/OHT: 66, Control: 15).

Uveoscleral outflow was found to be between 4% to 54% of total outflow [27,36]. One reason for the large variations are the measurement methods. Indirect methods estimate uveoscleral outflow using humor production rate, trabecular outflow resistance and $p_{EV}$, where $p_{EV}$ is not precisely known and has a significant impact on the result. Indirect methods result then in larger uveoscleral outflow than direct methods. Direct measurements in the human eye suggest a value of less than 15% [27,36]. Age-related decrease in uveoscleral flow was observed. A value of 1.52 mL/min was reported in eyes aged 20–30 years compared with 1.10 mL/min in eyes older than 60 years [27]. Similar to the ocular AH production rate, uveoscleral outflow is relatively pressure-independent in the normal IOP range [27].

## Appendix C. Step-by-Step Procedure for a Hexagonal Glaucoma Stent Design

**Table A1:** The step-by-step procedure for personalized stent design.

Steps	Input	Input	Output	Equation
1	$IOP_{BS}$ measured before surgery, e.g., 25 mmHg	$IOP_{AS}$ targeted after surgery, e.g., 14 mmHg	Serial outflow resistance $R_{sa}$ of stent and bleb array	$R_{sa}=\frac{IOP_{AS}\left(IOP_{BS}-p_{EV}\right)}{\left(\dot{Q}-{\dot{Q}}_{UP}\right)\left(IOP_{BS}-IOP_{AS}\right)}$ Equation (16), e.g., $\left(\dot{Q}-{\dot{Q}}_{UP}\right)=2.1\frac{\mu L}{\min}$
2	Hemispherical bleb radius for COMSOL simulation, e.g., $r_{b}=30\;\mu m$	Bleb spacing in array for COMSOL simulation, e.g., $d_{a}=300\;\mu m$	Bleb array drainage resistance $R_{a}$	$R_{a}=p_{b}/{\dot{Q}}_{s}$ where $p_{b}$ is the pressure in the blebs from COMSOL simulation and ${\dot{Q}}_{s}$ is the flow rate through the stent, e.g., ${\dot{Q}}_{s}=1.7\frac{\mu L}{\min}$ $R_{a}>\frac{4\times 10^{4}\left[\mathrm{mmHg}\frac{\min}{\mu L}\cdot \mu m\right]}{n\cdot r_{b}\left[\mu m\right]}$ Equation (13), where n is the number of outlets
3	$R_{sa}$ (from step 1)	$R_{a}$ (from step 2)	Stent flow resistance $R_{s}$	$R_{s}=R_{sa}-R_{a}$
4	Number of stent columns, e.g., $n_{c}=42$	Number of stent rows, e.g., $n_{r}=20$	Number of stent outlets and micro blebs	$n=n_{c}\cdot n_{r}$
5	Stent column resistance $R_{c}=n_{c}\cdot R_{s}$	Fix lowest stent outlet resistance, e.g., $R_{1}=R_{0}/10$	Stent flow resistance $R_{0}$ connecting two outlet tubes	$R_{0}=\frac{6\left(n_{r}R_{c}-R_{1}\right)}{n_{r}\left(3n_{r}-1\right)}=\frac{6\left(nR_{s}-R_{1}\right)}{n_{r}\left(3n_{r}-1\right)}$ Equation (5)
6	Hexagonal segment flow resistance $R=\frac{2}{3}R_{0}$	Bleb spacing $d_{a}$	Channel length L and cross section width D of hex. segment	$L=d_{a}\tan30{}^{\circ}$ (geometry of hexagon) D=28.4·ηLR14=42.7·ηLR014 Equations (1) and (2)
7	$R_{0}$ (from step 5)	Lowest stent outlet resistance $R_{1}$ (from step 5)	Flow resistance $R_{k}$ of outlet tube in row number k	$R_{k}=\frac{1}{2}k\left(k-1\right)R_{0}+R_{1}$ Equation (4)
8	Length $L_{K}$ of straight outlet tube, e.g., 100 μm	Row number k	Cross section width of outlet tube	Dk=28.4·ηLkRk14 Equation (3)

## Appendix D. List of Parameters Used for Calculations

**Table A2:** Governing parameters for calculations.

Parameter	Value	SI Units	Description
ϱ	$10^{3}$	$\frac{\mathrm{kg}}{m^{3}}$	Density of fluid, of AH
η	$7\times 10^{-4}$	Pa·s	Dynamic viscosity of liquid
K	$5\times 10^{-13}$	$\frac{m^{2}}{\mathrm{Pa}\cdot s}$	Hydraulic conductivity in subconjunctival tissue [6]
κ=K·η	$3.5\times 10^{-16}$	$m^{2}$	Fluid permeability in subconjunctival tissue, used in Equation (6)
$S_{p}=\varrho \cdot L_{p}\frac{S_{A}}{V}$	$10^{-3}$	$\frac{s}{m^{2}}$	Used in Equation (6)
$L_{p}$	$10^{-10}$	$\frac{m}{\mathrm{Pa}\cdot s}$	Hydraulic permeability of blood vessel wall [6]
$\frac{S_{A}}{V}$	$10^{4}$	$\frac{1}{m}$	Vessel wall area per tissue volume [6]
$\frac{1}{\sqrt{C}}=\sqrt{\frac{\varrho }{S_{p}}\frac{\kappa }{\eta }}$	$7\times 10^{-4}$	m	Characteristic drainage length, used in Equation (8)
$\dot{Q}$	2.5	$\frac{\mu L}{\min}$	Typical AH production rate
${\dot{Q}}_{UP}≅0.15\cdot \dot{Q}$	0.4	$\frac{\mu L}{\min}$	Constant outflow rate through uveoscleral pathway
$p_{EV}$	11	mmHg	Episcleral venous pressure, ranging from 9 to 12 mmHg [6]
